# Plasmocytome solitaire pulmonaire traité par radiothérapie: à propos d’un cas et revue de la littérature

**DOI:** 10.11604/pamj.2019.34.92.20089

**Published:** 2019-04-23

**Authors:** Badr Elmorabit, Najib Derhem, Mouna Khouchani

**Affiliations:** 1 Service d’Oncologie Radiothérapie, Centre Hospitalo-Universitaire Mohammed VI, Avenue Ibn Sina, Marrakech, Maroc

**Keywords:** Plasmocytome extramédulaire, plasmocytome pulmonaire, plasmocytome solitaire, Extramedullary plasmacytoma, plasmacytoma of the lung, solitary plasmacytoma

## Abstract

Les proliférations plasmocytaires malignes extra-médullaires isolées qu'on appelle plasmocytomes sont rares. Elles sont décrites habituellement au niveau de la tête et du cou (80% des cas) et exceptionnellement ailleurs. Nous rapportons l'observation d'un patient de 62 ans qui présente un plasmocytome solitaire pulmonaire mimant initialement un cancer bronchique primitif. Nous rapportons notre attitude thérapeutique et discutons à travers une revue de la littérature la rareté de ce cas clinique, les facteurs pronostiques ainsi que les modalités de prise charge de ces tumeurs.

## Abstract (en)

Isolated malignant extramedullary plasma cell proliferations known as plasmocytomas are rare. They usually occur in the head and the neck (80% of cases) and exceptionally elsewhere. We report the case of a 62-year old patient with solitary plasmacytoma of the lung initially mimicking primary lung cancer. The purpose of this study was to describe our therapeutic approach and to emphasize the rarity of this case, the prognostic factors as well as the management of these tumors based on a literature review.

## Introduction

Le plasmocytome solitaire est une tumeur maligne rare appartenant à la famille des proliférations plasmocytaires. Il est défini comme une tumeur plasmocytaire maligne isolée, localisée sans signes de dissémination. Nous distinguons les plasmocytomes osseux (PO) qui sont les plus fréquents et les plasmocytomes extra osseux ou extra-médullaires (PEM) beaucoup plus rares (3% des proliférations plasmocytaires) dont 80% sont localisés au niveau des voies respiratoires supérieures [1]. L'atteinte pulmonaire primitive est exceptionnelle (moins de 2% des PEM) [1]. La radiosensibilité et la radiocurabilité des plasmocytomes solitaires ont été démontrées et la radiothérapie et la chirurgie constituent les traitements de référence [2]. Nous rapportons une nouvelle observation de PEM pulmonaire et tentons de dégager les principales caractéristiques de cette entité à travers une revue de la littérature portant sur des observations similaires précédemment colligées.

## Patient et observation

**Figure 1:**
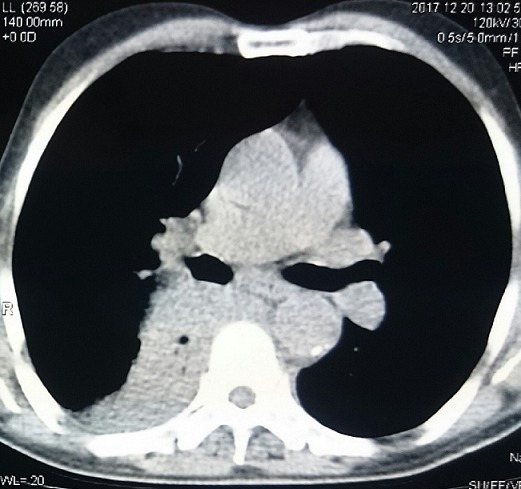
Scanner thoracique montrant un processus tumoral lobaire inférieur droit

Un patient de 62 ans, tabagique chronique, sans autres antécédents pathologiques particuliers, consulte pour une toux sèche évoluant depuis 4 mois associée à une dyspnée d'effort d'aggravation progressive. La radiographie du thorax a montré une opacité lobaire inférieure droite. La tomodensitométrique thoracique faite par la suite a objectivé un processus tumorale du lobe inférieure droit de 48x42 mm localisé en paravertébrale droit (Figure 1). La bronchoscopie a mis en évidence un aspect inflammatoire de 1^er^ degré des bronches droites sans tumeur visible. Il a été décidé de faire une biopsie pulmonaire scannoguidée. Cette dernière a montré une prolifération tumorale à cellules rondes à différenciation majoritairement plasmocytoïde. L’étude immunohistochimique a montré une positivité des anticorps anti CD138, EMA et anti KI67 et une négativité des anti chromogranine et synaptophysine. Biologiquement, on a noté une augmentation des gammaglobulines en rapport avec l'existence d'un pic monoclonal discret de type IgM. La fonction rénale et la b2 microglobuline sérique sont normales. Le myélogramme, la biopsie ostéomédullaire et le bilan radiologique osseux sont sans anomalies. Il a été décidé de faire une radiothérapie exclusive à 50Gy sur la masse tumorale (2Gy/séance, 5 séances par semaine) en conformationelle. L'évolution a été marquée par une nette amélioration clinique avec disparition du pic monoclonale et une diminution de 60% de la taille tumorale au scanner de contrôle à 3 mois. Avec un recul de 18 mois on a noté une stabilité de la masse tumorale sans argument en faveur d'une récidive ou d'une progression vers un myélome multiple.

## Discussion

Le plasmocytome extramédullaire est une prolifération monoclonale de plasmocytes dans les tissus mous ou dans un organe. La relation entre le myélome multiple (MM), PO solitaire et PEM n'est pas bien comprise. Pour certains auteurs ces 3 entités représentent différents aspects de la même maladie. D'autres considèrent le plasmocytome solitaire de l'os comme une manifestation rare du myélome multiple. Le PEM doit cependant être considéré différemment rejoignant les tumeurs solides qui peuvent infiltrer les ganglions lymphatiques à proximité ou provoquer des métastases à distance [3]. Etienne *et al.* [4] qui ont pris en considération les critères diagnostiques suivants: un prélèvement anatomopathologique typique, une atteinte pulmonaire parenchymateuse ou bronchique inaugurale et l'absence d'atteinte médullaire, ont répertorié 37 cas de PEM pulmonaires rapportées sur une période de 40 ans. Depuis lors 10 autres nouveaux cas ont été publiés [5-12]. Les principales caractéristiques de ces patients sont détaillées dans le Tableau 1 [4-12]. Les PEM pulmonaires se caractérisent par un âge médian au diagnostic de 59 ans, un pic de fréquence au cours de la septième décennie avec un sex-ratio proche de 2/1. La symptomatologie clinique étant variée et peu spécifique pouvant mimer une tumeur primitive pulmonaire. Elle met en évidence le plus souvent une opacité nodulaire parenchymateuse, parfois périhilaire, plus ou moins bien limitée, associée à d'éventuelles adénopathies médiastinales. Les localisations tumorales multiples, les atteintes alvéolo-interstitielles diffuses ou endobronchiques ne sont que rarement rapportées [4]. Le diagnostic repose, d'une part sur l'absence d'argument en faveur d'un myélome et d'autre part, sur l'examen anatomopathologique d'une pièce opératoire, de biopsies transpariétales, de biopsies transbronchiques, plus rarement sur l'étude cytologique du lavage bronchoalvéolaire. Cet examen anatomopathologique montre une prolifération cellulaire en nappe dont les caractéristiques morphologiques peuvent déjà orienter vers l'origine plasmocytaire.

**Tableau 1:** Plasmocytomes extra médullaires pulmonaires (n=47)

Sexe (H/F)		32/15
Age médian		57,3(14-89)
Présentation radiologique	Localisation tumorale unique	37(78%)
	Localisations tumorales multiples	10(22%)
Gammapathie monoclonale sérique (n=37)	Totale	16(34%)
	IgG	13(27,6%)
	IgA	3(6,3%)
Traitement	Chirurgie	21(44,6%)
	Radiothérapie	4(8,5%)
	Chirurgie + radiothérapie	8(17%)
	Chimiothérapie	8(17%)
	Chirurgie + chimiothérapie	3(6,3%)
	Chimiothérapie + radiothérapie	2(4,2%)
	Laser	1(2%)
Suivi médian (mois)		18 mois
Évolution	Réponse complète-contrôle local	31(65%)
	Récidive locale	2(5%)
	Récidive autre site	6(14%)
	Myélome multiple	6(14%)
	Décès	13(30%)

L'étude immunohistochimique vient généralement confirmer le diagnostic et les distinguer des lymphomes malins non hodgkiniens de bas grade à différenciation plasmocytaire. Elle va préciser la nature de l'immunoglobuline intracytoplasmique exprimée (G ou A, ou restreinte à une seule chaîne légère kappa ou lambda) et l'absence d'expression d'antigènes lymphocytaires B tels que le CD20 [13]. La signification pronostique d'un pic de gammapathie monoclonale reste incertaine. La nature de celle-ci (de type non IgG), la persistance de celle-ci après traitement semblent corrélées au risque d'évolution vers le MM [5,14]. Le plasmacytome pulmonaire primaire évolue différemment du myélome multiple et des taux de survie prolongés ont été décrits. En effet d'après l'analyse des cas colligés dans le Tableau 1 [4-12], nous constatons que malgré des attitudes thérapeutiques disparates, 65% des patients n'ont présenté ni récidive ni MM, 19% ont eu une rechute locale ou à distance. L'évolution vers un MM a été constatée dans 14% des cas et le plus souvent dans les deux à trois ans suivant le diagnostic de PEM pulmonaire. Des résultats comparables ont été retrouvés par Alexiou *et al.* Dans cette étude rétrospective portant sur 869 cas de PEM (82% de PEM des voies aérodigestives supérieures (VADS), 18% de PEM d'autres localisations) montrant des taux de récidive et de survenue d'un MM respectivement de 22 et 16,1% pour les PEM des VADS et de 21,2 et 14,1% pour les PEM d'autres localisations [1]. Une étude plus récente de Katodritou *et al.* a examiné 97 patients atteints de plasmocytomes (PEM et PO) et a rapporté que 25% (24/97) des cas ont évolué vers un MM [15]. D'autres études ont suggéré que ces patients avec PEM ont un risque de progression vers un MM relativement faible par rapport aux patients avec PO [12]. Il n'existe pas encore de consensus bien établi sur la prise en charge des PEM en générale et le plasmocytome pulmonaire en particulier. L'exérèse chirurgicale peut être considérée comme curative à elle seule si on arrive à avoir des marges saines [16].

Un certain nombre de patients traités chirurgicalement ont eu également une radiothérapie adjuvante. La radiothérapie seule constitue également un des traitements de référence du plasmocytome solitaire extramedullaire et du plasmocytome solitaire osseux, soit exclusivement, soit après une chirurgie à visée essentiellement diagnostique. Elle a une efficacité comparable à la chirurgie et permet d'assurer le contrôle local dans 70 à 100% des cas [17]. La dose recommandée varie selon les auteurs, elle est de 45Gy à 50Gy (en cas de tumeur volumineuse) [17,18]. Tournier *et al.* ont rapporté dans une série de 17 malades suivis pour plasmocytome extramédullaire de la tête et du cou, un taux de contrôle local à cinq ans de 100% chez neuf patients ayant reçu une dose d'au moins 45Gy dans le volume anatomoclinique (CTV) contre 50% chez huit patients ayant reçu une dose de moins de 45Gy (p=0,0034) [17]. De même, un effet de dose a été mentionné par Mendenhall *et al.* qui ont rapporté un taux de contrôle local de 94% à des doses supérieures à 40Gy contre 69% dans le cas contraire [19]. En plus de l'effet dose, qui est le facteur principal prédictif en termes de contrôle local, d'autres facteurs ont été étudiés dans la littérature. Tsang *et al.* ont trouvé que la taille tumorale constituait le premier facteur influençant la réponse locale à la radiothérapie avec un taux de réponse complète de 100% si elle était de moins de 5 cm et 38% dans le cas contraire [20]. Dans la série de Tournier *et al.* le siège de la tumeur initiale, la destruction osseuse, un pic monoclonal d'immunoglobuline n'avaient pas un impact sur le contrôle local [17]. La place de la chimiothérapie dans le traitement des PEM reste à définir, l'expérience étant relativement limitée. Par analogie aux chimiothérapies du MM, les agents alkylants (melphalan, cyclophosphamide) ont été le plus souvent utilisés en association à la prednisone. Elle a été proposée dans les formes diffuses, les localisations multiples et les récidives. L'analyse de la littérature médicale nous pousse à penser que le plasmocytome pulmonaire primitif se comporte de la même manière que les autres plasmocytomes extramédullaires qui ont tendance à infiltrer des ganglions lymphatiques régionaux ou à métastaser à des sites situés en dehors du thorax, mais sans envahir forcément la moelle osseuse [8]. Si cette interprétation sera confirmée, la gestion de la maladie consistera principalement en un traitement local (chirurgie ou radiothérapie) visant à prévenir leur invasion et leur propagation locale. Pour les tumeurs de plus de 5 cm ou très indifférenciées, il conviendrait d'évaluer l'intérêt d'une chimiothérapie adjuvante [16].

## Conclusion

Le plasmocytome pulmonaire solitaire reste une entité très rare des plasmocytomes extramedullaires. Le tableau clinique est souvent celui d'un cancer primitif du poumon. La radiothérapie avec une dose minimale de 45Gy constitue une option thérapeutique efficace et équivalente à la chirurgie. Notre cas semble être traité avec succès par la radiothérapie seule sans signes de récidive après un recul de 18 mois. Le risque de rechute locale et à distance et la transformation en MM justifient une surveillance régulière.

## Conflits d’intérêts

Les auteurs ne déclarent aucun conflit d'intérêts.
